# Wild Artichoke (*Cynara cardunculus* subsp. *sylvestris,* Asteraceae) Leaf Extract: Phenolic Profile and Oxidative Stress Inhibitory Effects on HepG2 Cells

**DOI:** 10.3390/molecules28062475

**Published:** 2023-03-08

**Authors:** Rosaria Acquaviva, Giuseppe Antonio Malfa, Rosa Santangelo, Simone Bianchi, Francesco Pappalardo, Maria Fernanda Taviano, Natalizia Miceli, Claudia Di Giacomo, Barbara Tomasello

**Affiliations:** 1 Department of Drug and Health Sciences, University of Catania, Viale A. Doria 6, 95125 Catania, Italy; 2 Research Centre on Nutraceuticals and Health Products (CERNUT), University of Catania, Viale A. Doria 6, 95125 Catania, Italy; 3 *PLANTA*/Autonomous Center for Research, Documentation and Training, Via Serraglio Vecchio 28, 90123 Palermo, Italy; 4 Department of Chemical, Biological, Pharmaceutical and Environmental Sciences, University of Messina, Viale Ferdinando Stagno d’Alcontres 31, 98166 Messina, Italy

**Keywords:** polyphenols, cynarine, Nrf2, glutathione, ROS, cytokines, hepatoprotection

## Abstract

*Cynara cardunculus* subsp. *sylvestris* (wild artichoke) is widespread in Sicily, where it has been used for food and medicinal purposes since ancient times; decoctions of the aerial parts of this plant have been traditionally employed as a remedy for different hepatic diseases. In this study, the phenolic profile and cell-free antioxidant properties of the leaf aqueous extract of wild artichokes grown in Sicily (Italy) were investigated. The crude extract was also tested in cells for its antioxidant characteristics and potential oxidative stress inhibitory effects. To resemble the features of the early stage of mild steatosis in humans, human HepG2 cells treated with free fatty acids at the concentration of 1.5 mM were used. HPLC-DAD analysis revealed the presence of several phenolic acids (caffeoylquinic acids) and flavonoids (luteolin and apigenin derivatives). At the same time, DPPH assay showed a promising antioxidant power (IC_50_ = 20.04 ± 2.52 µg/mL). Biological investigations showed the safety of the crude extract and its capacity to counteract the injury induced by FFA exposure by restoring cell viability and counteracting oxidative stress through inhibiting reactive oxygen species and lipid peroxidation and increasing thiol-group levels. In addition, the extract increased mRNA expression of some proteins implicated in the antioxidant defense (Nrf2, Gpx, and SOD1) and decreased mRNA levels of inflammatory cytokines (IL-6, TNF-α, and IL-1β), which were modified by FFA treatment. Results suggest that the total phytocomplex contained in wild artichoke leaves effectively modulates FFA-induced hepatic oxidative stress.

## 1. Introduction

*Cynara* L. (Asteraceae) is a small genus native to the Mediterranean area. Only a few taxa comprise this genus, including wild artichoke (*Cynara cardunculus* (L.) subsp. *sylvestris* Lam.), which is considered the wild progenitor of the globe artichoke (*Cynara cardunculus* (L.) subsp. *scolymus* (L.) Hegi) and of the leafy or cultivated cardoon (*Cynara cardunculus* (L.) var. *altilis* DC.) [1,2]. Artichoke is an edible and medicinal plant with a long tradition of use, which dates to the ancient Egyptians, Greeks, and Romans [3]. The edible part of both wild and cultivated species consists of the immature inflorescences (capitula or heads), which are consumed as a vegetable worldwide [4], whereas the leaves are traditionally used as a herbal remedy against liver complaints as hepatoprotective, choleretic, diuretic, and lipid-lowering agents [1,5,6,7]. Recently, the Committee on Herbal Medicinal Products (HMPC) of the European Medicines Agency (EMA) recognized preparations from *C. cardunculus* leaves as herbal remedies for the symptomatic relief of digestive disorders [8].

*Cynara cardunculus* subsp. *sylvestris* (*C. sylvestris*) is a robust perennial plant with wide, spiny leaves that form a large characteristic rosette in winter, showing a branched flowering stem in late spring and blue-violet flowers [9,10]. It is native to the Mediterranean Basin, and it is also found in south Portugal, in the Canary Islands, and in the Azores Islands [2]. In post-Columbian times, wild artichoke colonized parts of the New World, and it is now a weed in some areas of Argentina, Chile, and California [10]. Wild artichoke is widely present in Sicily (Italy), where it has been used for food and medicinal purposes since ancient times. Despite of this, the potential of wild Sicilian artichoke as a source of biologically active compounds is still underexplored; indeed, only few studies about the phenolic composition of different plant parts have been carried out [11,12]. *Cynara cardunculus* taxa have been shown to be a rich source of a large variety of active phytochemicals; most of the biological activities reported are ascribed to the phenolic compounds contained in the different plant organs, represented by various classes, namely hydroxycinnamic acid derivatives and flavonoid derivatives [5,13,14]. Among them, *C. cardunculus* subsp. *scolymus*, the globe artichoke, has been studied extensively due to its economic importance; indeed, in addition to its nutritional and phytochemical interest, this species is utilized for several industrial applications [4,5,13,15]. Due to their long-standing medicinal uses, artichoke leaves have been the subject of several investigations; many of these have focused on the potential of artichoke leaf extracts for liver protection [13,14,16]. Recently, the positive benefits on liver steatosis (NAFLD) have been demonstrated both in preclinical studies and in clinical trials for artichoke extracts, alone or in combination with nutraceuticals; a few of these studies highlighted the beneficial effects of wild artichoke on liver [17,18,19,20,21].

Both the two-hit and the newer multiple parallel hits theory on NAFLD inflammatory and fibrotic progression hypothesize the involvement of oxidative stress in free fatty acid (FFA)-induced lipotoxic liver injuries [22,23]. In this context, the present work aimed at deepening the knowledge of wild artichoke leaves as valuable sources of bioactive compounds helpful in counteracting oxidative stress conditions in an in vitro model of early stages of liver steatosis.

For the current study, the leaves of *C. cardunculus* subsp. *sylvestris* grown in Sicily were utilized; an aqueous extract was prepared, with water being the solvent commonly utilized for traditionally used artichoke herbal preparations [8]. The phenolic compounds contained in the extract were characterized via HPLC-DAD analysis. The antioxidant ability was established in vitro via the DPPH test, and the antioxidant effects were evaluated in steatotic HepG2 cells via the determination of ROS thiol groups (RSH), of lipid peroxides (LOOH), of mRNA expression levels of antioxidant defense markers (Nrf2, Gpx, and SOD1), and of inflammatory cytokines (TNF-α, IL-1β, and IL-6). The potential cytotoxicity of the extract was defined by an MTT test on HepG2 cells and by the *Artemia salina* lethality assay.

## 2. Results

### 2.1. Phytochemical Analysis

**Figure 1:**
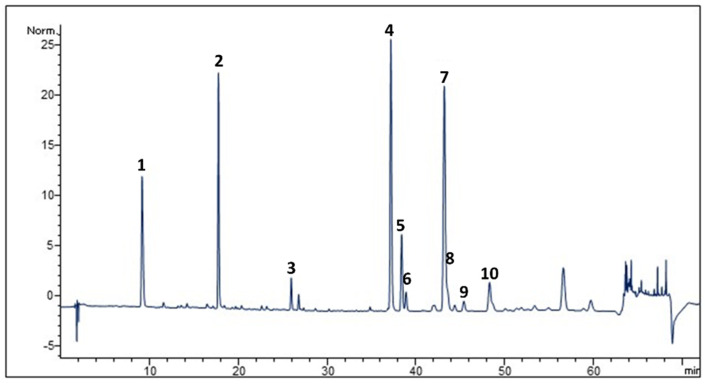
HPLC-DAD polyphenol fingerprint of *Cynara sylvestris* leaf extract. Column: Ascentis Express C18, 15 cm × 4.6 mm, 2.7 µm d.p. For peak identification, see Table 2.

The total flavonoid content (TFC) and the total phenolic content (TPC) of *C. sylvestris* extract were equal to 50.32 ± 1.62 mg catechin equivalent (CE)/g extract and 185.21 ± 1.97 mg gallic acid equivalent (GAE)/g extract, respectively (Table 1). The phytochemical profile obtained via HPLC-DAD revealed the presence of several phenolic acids (caffeoylquinic acids) and flavonoids such as luteolin and apigenin derivatives (Figure 1, Table 2).

### 2.2. Cell Viability

**Figure 2:**
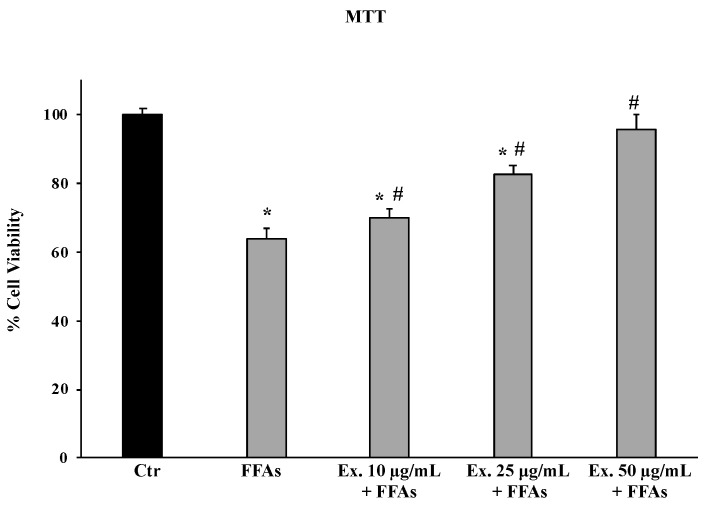
Cell viability in HepG2 cells untreated (Ctr), HepG2 cells treated for 12 h with 1.5 mM FFAs 1, and HepG2 cells pre-treated with the extract (10–25–50μg/mL) and exposed to FFAs 1. Values are the mean ± S.D. of three independent experiments in triplicate. * Significant vs. untreated control cells: *p* < 0.001. # Significant vs. FFAs treated cells: *p* < 0.001.

The MTT assay demonstrated that treatment for 24 h of *C. sylvestris* leaf extract at different concentrations (10–50 μg/mL) is not cytotoxic for HepG2 cells (data not reported); in contrast, treatment of HepG2 cells for 12 h with FFAs [1.5 mM oleic acid and palmitic acid (2:1)], which reproduces an in vitro model of early stages of liver steatosis, reduces viability by approximately 40%. Pre-treatment, on the other hand, with the extract for 24 h can counteract the damage caused by FFA exposure. Particularly, the cells pre-treated with 50 µg/mL of extract showed a cell viability value comparable to that recorded in control cells (Figure 2).

### 2.3. Cell-Free Antioxidant Properties

The antioxidant activity of *C. sylvestris* was assessed via the DPPH test. As reported in Table 1, the extract exhibited a concentration-dependent quenching effect with an IC_50_ value of 20.04 ± 2.52 µg/mL, which is equivalent to 15 μM ± 0.62 of Trolox.

### 2.4. Cell-Based Antioxidant Properties

#### 2.4.1. *Cynara sylvestris* Extract Reduced Reactive Oxygen Species Levels

**Figure 3:**
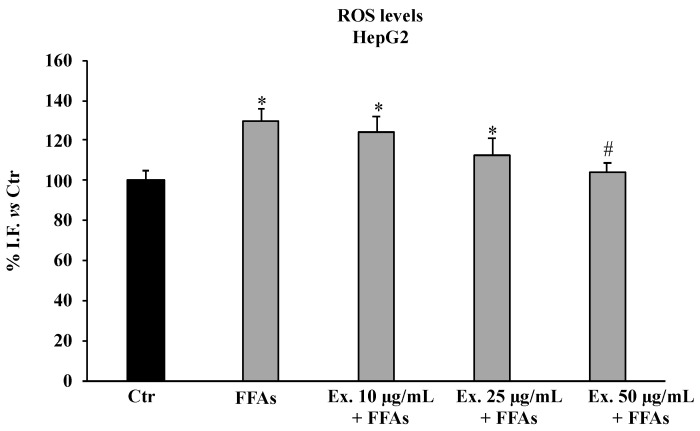
ROS production in HepG2 untreated cells (Ctr), HepG2 cells treated for 12 h with 1.5 mM FFAs 1, and HepG2 cells pre-treated with the extract (10–25–50 μg/mL) and exposed to FFAs 1. Results are expressed as percentage of intensity of fluorescence (I.F.)/mg protein vs. Ctr. Values are the mean ± S.D. of three independent experiments in triplicate. * Significant vs. untreated control cells: *p* < 0.001. # Significant vs. FFAs treated cells: *p* < 0.001.

Figure 3 shows that FFA treatment significantly increased ROS levels, while the pre-treatment with the extract significantly counteracted ROS production only at the concentration of 50 μg/mL.

#### 2.4.2. *Cynara sylvestris* Extract Reduced Lipid Hydroperoxide Levels

**Figure 4:**
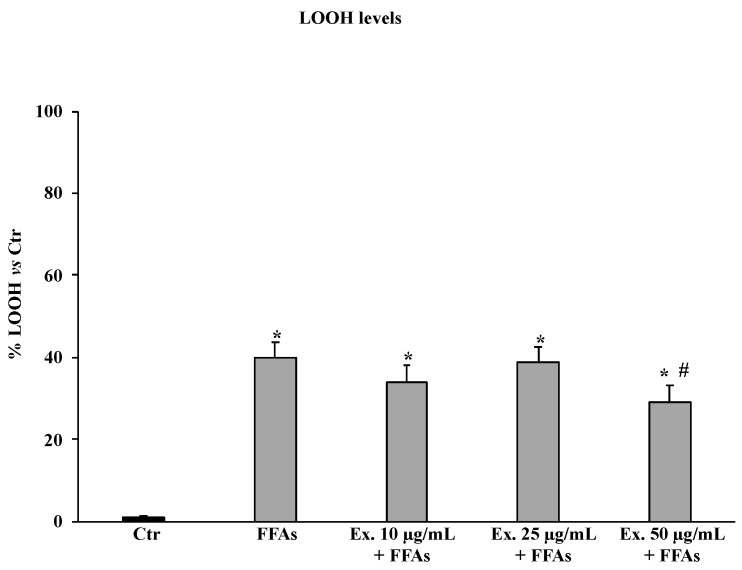
LOOH levels in HepG2 untreated cells (Ctr), HepG2 cells treated for 12 h with 1.5 mM FFAs, and HepG2 cells pre-treated with the extract (10–25–50 μg/mL) and exposed to FFAs 1. Results are expressed as percentage of lipid hydroperoxide levels vs. Ctr. Values are the mean ± S.D. of three independent experiments in triplicate. * Significant vs. untreated control cells: *p* < 0.001. # Significant vs. FFAs treated cells: *p* < 0.001.

The exposure of HepG2 cells with FFAs for 12 h induced an increment in LOOH levels compared to untreated controls; pre-treatment with extract exerted a protective effect only at the concentration of 50 μg/mL (Figure 4).

#### 2.4.3. *Cynara sylvestris* Extract Preserved Total Thiol Group Levels

**Figure 5:**
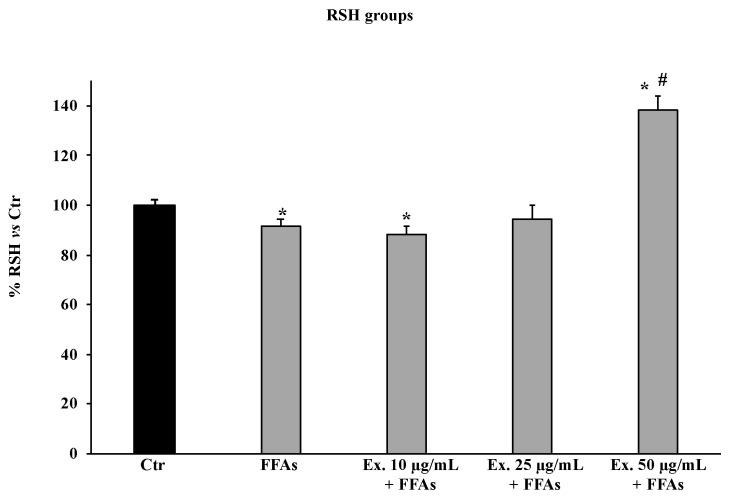
RSH levels in HepG2 untreated cells (Ctr), HepG2 cells treated for 12 h with 1.5 mM FFAs 1, and HepG2 cells pre-treated with the extract (10–25–50 μg/mL) and exposed to FFAs 1. Results are expressed as percentage of total thiol group levels vs. Ctr. Values are the mean ± S.D. of three independent experiments in triplicate. * Significant vs. untreated control cells: *p* < 0.001. # Significant vs. FFAs treated cells: *p* < 0.001.

The exposure of HepG2 cells to FFAs slightly reduced RSH levels compared to untreated cells, and interestingly, RSH content was improved only by the pre-treatment with the extract at 50 μg/mL; at this concentration, RSH levels were higher than in control cells (Figure 5).

#### 2.4.4. Effect of *Cynara sylvestris* Extract on Nrf2, Gpx, SOD1 mRNA Expression Levels

**Figure 6:**
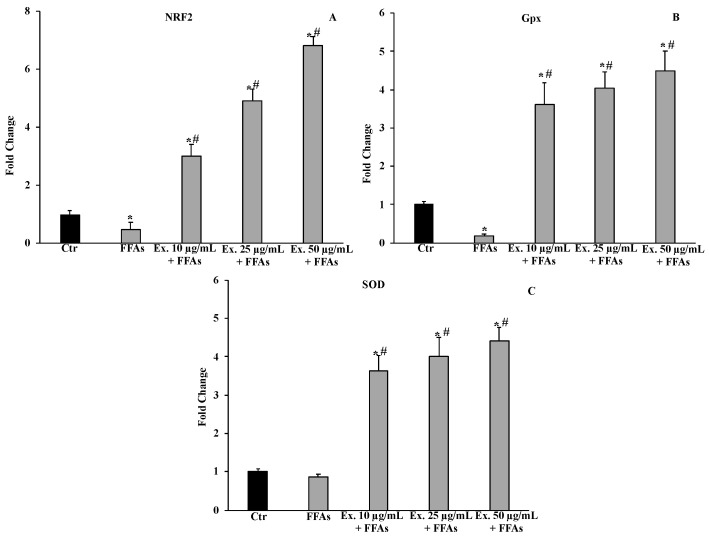
Effects of *Cynara sylvestris* extract pretreatment (10–25–50 µg/mL) for 24 h on mRNA expression levels of oxidative stress related genes: Nrf2 (nuclear factor erythroid 2–related factor 2) (**A**), Gpx (Glutathione peroxidase) (**B**), and SOD1 (Superoxide dismutase) (**C**). The data are reported as the mean ± S.D. of three independent experiments in triplicate. * Significant vs. untreated control cells *p* < 0.001. # Significant vs. FFAs, *p* < 0.001.

As depicted in Figure 6A–C, exposure to FFAs markedly reduced mRNA expression levels of Nrf2 and Gpx, while the pre-treatment with *C. sylvestris* extract dose-dependently increased all examined genes, including SOD1, which was not affected by FFAs exposure.

#### 2.4.5. Effect of *Cynara sylvestris* Extract on TNF-α, IL-6, and IL-1β mRNA Expression

**Figure 7:**
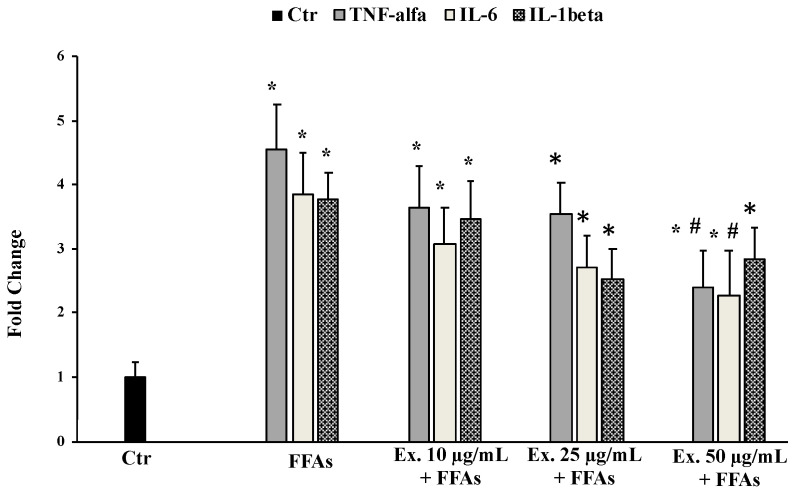
Effects of *Cynara sylvestris* extract pretreatment (10–25–50 µg/mL) for 24 h on mRNA expression levels of oxidative-stress-related genes: TNF-alfa, IL-6, and IL-1 beta. The data are reported as the mean ± S.D. of three independent experiments in triplicate. * Significant vs. untreated control cells *p* < 0.001. # Significant vs. FFAs, *p* < 0.001.

Figure 7 shows that the increased mRNA expression of inflammatory cytokines (IL-6, TNF-α, and IL-1β), which was induced by FFAs exposure, was partially counteracted by the pre-treatment with the extract.

#### 2.4.6. *Artemia salina* Lethality Bioassay

The evaluation of the toxic action of plant extracts is essential to consider a treatment safe; Artemia salina Leach is a small aquatic crustacean routinely used to perform different tests for preliminary toxicity estimation. Brine shrimp lethality is a short-term biological assay widely utilized for plant extracts in order to determine their potential toxicity [24]. The main advantages of using *A. salina* are the possibility of continuous supply and long storage of the cysts, the ease of keeping samples in laboratory conditions, and the inexpensive culture requirements [25]. After treatment with *C. sylvestris* leaf extract for 24 h, the A. salina larvae were all alive, even at the highest tested concentration of 1000 µg/mL. The results of this assay indicate the potential safety of the extract according to Clarkson’s toxicity criterion, which classifies plant extracts with an LC_50_ (median lethal concentration) above 1000 μg/mL as non-toxic [24].

## 3. Discussion

ROS production is an indirect result of aerobic metabolism; however, an increase in prooxidant products, when associated with an impairment in the endogenous antioxidant system, leads to a condition of oxidative stress. This harmful state is frequently observed during the inflammatory process of tissues and is involved in the etiology of most non-communicable diseases, including NAFLD and NASH [26,27,28]. The latest multiple parallel hits theory on NAFLD pathogenesis confirmed the role of oxidative stress in the lipotoxic liver injuries caused by FFAs, which was first hypothesized in the two hits theory [23]. Several studies have demonstrated that different classes of plant secondary metabolites can prevent and ameliorate oxidative stress in both physiological and pathological conditions and have highlighted that a polyphenol-rich diet exerts protective effects against the initiation and progression of different non-communicable and infectious diseases [29,30,31]. The beneficial effects of polyphenols are mainly attributable to their antioxidant activities, which are responsible for reducing chronic inflammation of tissues and preventing cell damage, even at the molecular level.

*Cynara cardunculus* taxa contain considerable quantities of polyphenols, mainly caffeoylquinic acid, apigenin, and luteolin derivatives. In this study, the crude aqueous extract from leaves of *C. sylvestris* grown in the wild in Sicily showed a significant amount of phenolic compounds (185.21 ± 1.97 mg GAE/g), as determined spectrophotometrically, of which slightly less than 30% is represented by flavonoids (50.32 ± 1.62 mg QE/g) (Table 1). The above results comply with a previous study in which a higher amount of polyphenols was found in the leaves of wild artichoke than in cultivated varieties [32]. Moreover, the highest content of phenolic compounds in that study was found in a leaf sample of wild artichoke from the same area of Sicily (Syracuse, Italy), where the leaves of this study were collected. The phytochemical profile determined via HPLC-DAD showed the presence of several phenolic acids, such as caffeoylquinic acid derivatives, including cynarine, and flavonoids, such as luteolin and apigenin derivatives (Figure 1), which are characteristic secondary metabolites of *C. cardunculus* spp. with broad antioxidant properties, as confirmed by the good free radical scavenging properties highlighted in the DPPH assay (IC_50_ 20.04 ± 2.52 µg/mL). These results confirmed the higher radical scavenger activity of wild artichoke with respect to the cultivated forms (globe artichoke) [33,34].

Starting from these extract characteristics and the absence of toxicity at the employed concentrations (Figure 2), the current study aimed to deepen existing knowledge of the protective effects of *C. sylvestris* extract on the redox state of human hepatoma cells in an in vitro model resembling the early stages of liver steatosis. In this model, HepG2 cells exposed for 12 h to FFAs at a total concentration of 1.5 mM exhibited a significant increase in ROS and LOOH levels and a slight decrease in RSH content. As previously reported, hepatocyte mitochondria increase beta-oxidation as a protective response to the presence of FFAs, with consequent enhancement of ROS production responsible for lipid peroxidation and depletion of intracellular glutathione [22,23]. In subjects with metabolic syndrome (type 2 diabetes or obesity), these cellular events add to ROS generated in the gut and adipose tissue, setting up a severe oxidative stress condition that leads to mitochondrial dysfunction and a gain in triglyceride (TG) accumulation in hepatocyte cytoplasm [22,23]. FFA-induced steatosis in HepG2 cells is associated with ROS production and consequent lipid peroxidation, which is also strictly related to the decrease in some antioxidant enzymes such as SOD1 and a concomitant increase in proinflammatory mediators including TNF-α [35,36].

The pre-treatment with the crude extract at 50 µg/mL significantly counteracted ROS production, shielding lipids from peroxidation and the intracellular RSH amount (Figure 3, Figure 4 and Figure 5). These results conform with previous studies carried out in different experimental models on the antioxidant effects of extracts from cultivated varieties of *C. scolymus* [37,38]. The antioxidative effects of plant secondary metabolites can be exerted via direct reaction with free radicals or indirectly at the molecular level by modulating the activity or expression of the intracellular enzymes involved in promoting or counteracting oxidative stress [39]. Particularly, phenolic compounds are capable of inducing the activation of the Nrf2-antioxidant response element signaling pathway by increasing both activities and expressions of several antioxidant enzymes, including the superoxide dismutase (SOD) to convert O_2_^•^ into H_2_O_2_ and the glutathione peroxidase (GPX) to remove it [40]. Even at the molecular level, the pre-treatment for 24 h with the phytocomplex contained in the *C. sylvestris* leaf extract at all tested concentrations exerted its antioxidant effects in HepG2 cells exposed to FFAs by increasing the mRNA expression levels of the transcription factor Nrf2 (nuclear factor E2-related factor 2) in a dose-dependent way and the mRNA expression levels of the endogenous antioxidant enzymes SOD1 and GPX markedly. These results highlight that *C. sylvestris* extract exerts its protective effects both by its direct scavenger activity and indirectly by the regulation at molecular levels of the antioxidant cellular defenses. High levels of ROS such as H_2_O_2_ in hepatocytes that have been exposed to FFAs are directly correlated with a promotion of an inflammatory response mediated by pro-inflammatory mediators such as TNF-α, IL-1β, and IL-6, particularly TNF-α and ROS, which are positively promoted by each other [41] and are capable of inducing apoptotic cell death in steatotic hepatocytes [42]. In our experimental model, the exposure of HepG2 cells to FFAs significantly promoted mRNA expression levels by about three-fold with respect to the control cells for all the examined pro-inflammatory cytokines (TNF-α, IL-1β, and IL-6), but only the pre-treatment with the *C. sylvestris* extract at 50 µg/mL partially counteracted the deleterious consequence to FFA treatment (Figure 7). The protective effects on the cellular oxidative state and the inflammatory response in FFA-exposed HepG2 cells were clearly shown in the MTT assay, in which the pre-treatments with the different concentrations of *C. sylvestris* extract dose-dependently preserved cell viability; in particular, the highest concentration of 50 µg/mL definitely protected the mitochondrial functionality, which was compromised by the subsequent FFA exposure at about the level of control cells (Figure 2).

In conclusion, results obtained in these in vitro experiments showed that *C. sylvestris* aqueous leaf extract could act with different mechanisms of action, effectively counteracting oxidative stress induced by FFA, especially at the highest concentration of 50 µg/mL, both by increasing RSH levels and turning off ROS production and lipid peroxidation and also by beneficially modulating cytoprotective Nrf2/ARE-regulated genes, thus mitigating the inflammatory response. Our findings are supported by previous studies reporting significant antioxidant and hepatoprotective activities exerted by several polyphenols present in *Cynara* spp. such as chlorogenic acid, dicaffeoylquinic acid, luteolin, and apigenin derivatives [43,44,45,46,47]. These results suggest that the total phytocomplex contained in *C. sylvestris* leaf is a valuable source of bioactive compounds that are effective in counteracting transient or mild conditions of hepatic oxidative stress, adding new evidence to the previous studies on the hepatoprotective properties of taxa belonging to the *Cynara* genus. Thanks to the ease of growing this species in poor and stony soils without adding water, this species, which is not fully exploited, may constitute a sustainable economic resource for the natural health product industry.

## 4. Materials and Methods

### 4.1. Chemicals and Reagents

Analytic-grade organic solvents were purchased from VWR (Milan, Italy). UHPLC-grade water (18 mW), UHPLC-grade MeOH, and formic acid were obtained from Carlo Erba (Milan, Italy). 2,2-diphenyl-1-picrylhydrazyl (DPPH); xylenol orange; 2′,7-dichlorofluorescein diacetate (DCFH-DA); 2,2-dithio-bis-nitrobenzoic acid; tetrazolium salt; free fatty acids; oleic acid; and palmitic acid (purity ≥ 99%) were purchased from Sigma-Aldrich (Milano, Italy), except as otherwise specified.

### 4.2. Plant Collection and Extraction Procedure

**Figure 8:**
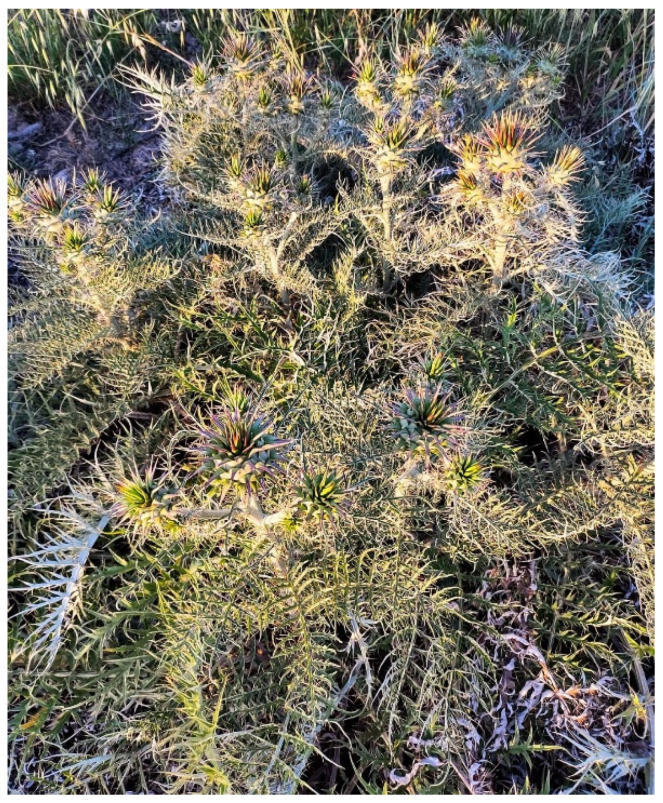
*Cynara cardunculus* (L.) subsp. *sylvestris* Lam. (Syracuse, Italy).

*Cynara sylvestris* was harvested in the seacoast area of Syracuse (Sicily, Italy) in May 2022 (Figure 8) and authenticated by botanist F.M. Raimondo. A voucher specimen (No. 05/22) was deposited in the herbarium of the Department of Drug and Health Sciences, Section of Biochemistry. After harvesting, fresh leaves were stored at −80 °C. An amount of 100 g of crushed plant material was extracted at 90 °C in water for 1 h (ratio 1:10). The extraction was repeated three times; then, the pooled solutions were filtered and evaporated to dryness with a rotatory evaporator, obtaining about 5.6 g of dry extract.

### 4.3. Determination of Total Flavonoid and Total Phenolic Content

In the extract, the content of TFC, which was analyzed spectrophotometrically, was compared with a calibration curve of a known quantity of catechin and expressed as mg of CE/g extract [48]. TPC was evaluated using the Folin–Ciocâlteu method [49]. The value obtained, compared with a calibration curve of a known quantity of gallic acid, was expressed as mg of GAE/g extract. Data were obtained from three independent determinations.

### 4.4. HPLC-DAD Analysis

High-pressure liquid chromatography (HPLC) was used to evaluate the polyphenolic fingerprinting of the extract as described above [50]. HPLC-DAD analyses were performed using a Shimadzu LC 20 (Kyoto, Japan), which was equipped with a diode array detector (DAD) and with a 150 × 4.6 mm i.d., 2.7 μm Ascentis Express C 18 column. The mobile phases were as follows: H_2_O/H_3_PO_4_ (99:1, solvent A), MeOH/CAN/H_3_PO_4_ (49,5:49,5:1, solvent B). The gradient used was as follows: concentration of the solvent A of 95% going to 77% (34 min), maintained at 77% (3 min), 74% (60 min), 60% (85 min), 20% (90 min), and 0% (92 min). The total time was 105 min. The column temperature was maintained at 25 °C. The flow was 1 mL/min, and the injection volume was 5 μL. The chromatogram profiles were recorded from 190 to 500 nm and monitored at 280 and 330 nm ± 2 nm.

### 4.5. DPPH Test

The DPPH test measures the quenching capacity of the extract spectrophotometrically at λ = 517 nm, as previously reported by Salerno [51]. The results, which were compared with Trolox (30 µM), were expressed as IC_50_ of the decrease in absorbance and represent the average ± S.D. of three independent experiments in triplicate.

### 4.6. Cell Culture and Treatments

The human hepatoma cell line (HepG2), which was obtained from ATCC^®^ HB-8065 (Rockville, MD, USA), was maintained in modified Eagle’s medium (MEM) supplemented with 10% *v/v* fetal bovine serum (FBS), 100 U/mL penicillin, and 100 µg/mL streptomycin.

HepG2 cells were cultured in a humidified atmosphere in 5% CO_2_ at 37 °C, and at sub-confluent conditions, they were plated at a constant density to obtain identical experimental conditions in the different tests.

### 4.7. MTT Assay

To evaluate cell viability, we used the MTT test, which measures the conversion of tetrazolium salt to yield colored formazan in the presence of metabolic activity. The amount of formazan is proportional to the number of living cells. The absorbance of the converted formazan was measured using a microplate spectrophotometer reader (Titertek Multiskan, Flow Laboratories, Helsinki, Finland) at λ = 570 nm. The results were presented as a percent of the control data [52]. HepG2 cells were treated for 12 h with FFAs (OA:PA at 2:1) at the total concentration of 1.5 mM, thereby resembling the features of mild steatosis in humans. To evaluate the protective effects of the extract, 24 h prior to the addition of FFAs, cells were pre-treated with *C. sylvestris* extract (10–25-50 µg/mL). The results are presented as a percentage of cell viability with respect to untreated control cells (100%).

### 4.8. Reactive Oxygen Species Assay

ROS levels were determined by using the 2′,7-dichlorofluorescein diacetate (DCFH-DA) fluorescent probe [53]. Briefly, differently treated and untreated HepG2 cells (1.5 × 10^5^ cells/mL) seeded in 6-well plates were incubated for 30 min with DCFH-DA (5 µM) at 37 °C. After cells were washed three times with PBS and treated with 1 mL of digitonin (2.5 mg/mL) at room temperature for 1 h and finally scraped, the obtained suspensions were centrifuged at 4 °C, 13,000× *g* for 10 min. Supernatants were used to determine ROS levels. Fluorescence (corresponding to oxidized radical species 2′,7′-dichlorofluorescein, DCF) was monitored spectrofluorimetrically (excitation, λ = 488 nm; emission, λ = 525 nm). Total protein content was evaluated for each sample, and the results are reported as a percentage of fluorescence intensity/mg of protein relative to the control. The protein content was determined using the Sinergy HTBiotech instrument by measuring the difference in absorbance at λ = 280 and λ = 260.

### 4.9. Lipid Peroxidation Determination

LOOH levels were evaluated via the oxidation of Fe^+2^ to Fe^+3^, which, in the presence of xylenol orange, produces the Fe^(3+)^-xylenol orange complex [53]. Differently treated and untreated HepG2 cells (1.5 × 10^5^ cells/mL) seeded in 6-well plates were scraped and washed three times with PBS. After centrifugation at 4 °C, 900× *g* for 10 min, the cellular pellet was resuspended in 500 μL of PBS and used to determine LOOH levels. The test was performed in a total volume of 1 mL; in particular, 200 μL of cellular suspension was added to 800 μL of a working solution of 100 μM xylenol orange, 250 μM ammonium ferrous sulfate, 4 mM butylated hydroxytoluene, and 25 mM H_2_SO_4_ in 90% methanol (V/V). After 30 min of incubation at room temperature, the absorbance at λ = 560 nm was measured using a U2000 Hitachi spectrophotometer (Tokyo, Japan). Calibration was obtained using hydrogen peroxide (0.2–20 μM). The results are expressed as a percentage of the increase with respect to the control (untreated cells).

### 4.10. Determination of Total Thiol Groups

Non-protein total thiol groups were determined using a spectrophotometric assay based on the reaction of thiol groups with 2,2-dithio-bisnitrobenzoic acid (DTNB) at λ = 412 nm [53]. In total, 200 μL of differently treated and untreated HepG2 cell suspension was added to 600 μL of TRIS base (0.25 M pH 8.2), followed by 400 μL of DTNB (10 mM) in absolute ethanol. After samples were incubated at room temperature for 20 min and centrifuged at 3000× *g* for 10 min at room temperature, the supernatants were used to determine total thiol group levels. Results are expressed as a percentage of the increase compared to the control (untreated cells).

### 4.11. RNA Extraction and Reverse Transcription-Quantitative Polymerase Chain Reaction

HepG2 cells (1.5 × 10^5^ cells/mL) seeded in 6-well plates were pretreated with *C. sylvestris* extract (10–25–50 mg/mL) for 24 h, followed by FFA exposure for 12 h. Total RNA was recovered by using TRIzol (Invitrogen, Carlsbad, CA, USA) and following the manufacturer’s instructions. Reverse transcription of 1 µg of total RNA was performed with a QuantiTect Reverse Transcription Kit (Qiagen Inc., Valencia, MD, USA). The cDNA obtained subsequently amplified by qPCR using the QuantiNova SYBR Green RT-PCR Kit according to the manufacturer’s instructions on Rotor-Gene Q5PLEX. Specific predesigned and bioinformatically validated primer sequences (QuantiTect Primer Assays, Qiagen Inc., Valencia, MD, USA) for inflammation genes TNFα (QT00029162), IL1β (QT00021385), and IL6 (QT00083720) and oxidative-stress-responsive genes Nrf2 (QT), SOD1 (QT01671551), and GPX (QT00203392) were used, according to previously described methods [54]. The expression levels of targets were normalized with RPLP0 (Ribosomal Protein Lateral Stalk Subunit P0) mRNA levels. Relative fold changes in gene expression were calculated using the 2^−ΔΔCt^ method.

### 4.12. Artemia Salina Lethality Bioassay

The toxicity of C. sylvestris leaf extract was also investigated in a living organism, i.e., *Artemia salina* Leach, by performing the lethality bioassay according to the protocol previously reported by Meyer et al. [55], with some modifications. *Artemia salina* cysts were placed in a hatchery dish containing artificial seawater (32 g sea salt/L) and incubated for hatching under a 60 W lamp, at a temperature of 24–26 °C. Twenty-four hours after hatching, ten brine shrimp larvae were placed in plates containing 5 mL of artificial seawater mixed with different volumes of the extract solution to obtain final concentrations in the range 10–1000 µg/mL, and the larvae were incubated at 24–26 °C for 24 h. At this time point, the surviving larvae were counted; then, LC_50_ (median lethal concentration) was estimated. Three replicates of each sample concentration were tested. Clarkson’s toxicity criterion was used to assess the toxicity level of the extract [24].

### 4.13. Statistical Analysis

Statistical analyses were performed by one-way and two-way ANOVA. The Bonferroni test was used as the post hoc test when the ANOVA reported statistically significant differences to evaluate the differences between the individual time-points or treatment groups. For all experiments, * *p* < 0.05 was considered to be significant.

## Figures and Tables

**Table 1:** Total polyphenols and flavonoids and DPPH-scavenging activity of *Cynara sylvestris* leaf extract.

	Total Flavonoids (mg CE/g Extract)	Total Polyphenols (mg GAE/g Extract)	DPPH Test IC_50_ (μg/mL)
*C. sylvestris*	50.32 ± 1.62	185.21 ± 1.97	20.04 ± 2.52 µg/mL
Trolox			15 µM ± 0.62

Values, which are expressed as mg gallic acid (GAE) and catechin (CE) equivalents, are the mean ± S.D. of three independent experiments in triplicate; IC_50_: half-maximal inhibitory concentration.

**Table 2:** Compounds in *Cynara sylvestris* leaf extract identified via HPLC-DAD.

Peak	Compound	Wavelength (nm)	Ret. Time (min.)
1	Neochlorogenic acid	325	9.11
2	Chlorogenic acid	325	17.79
3	Cynarine	325	25.92
4	Luteolin 7-Glucoside	346	37.21
5	Luteolin 7-Glucuronide	346	38.44
6	3,4-Dicaffeoylquinic acid	325	38.93
7	1,5-Dicaffeoylquinic acid	325	43.18
8	Apigenin 7-O-Rutinoside	346	43.45
9	Apigenin 7-O-Glucoside	346	45.38
10	Apigenin 7-O-Glucuronide	346	48.29

## Data Availability

The data presented in this study are available on request from the corresponding author.

## Sample Availability

Samples of the tested extract are not available from the authors.
